# Brain-derived neurotrophic factor in primary headaches

**DOI:** 10.1007/s10194-012-0454-5

**Published:** 2012-05-15

**Authors:** Marlene Fischer, Georg Wille, Stephanie Klien, Hind Shanib, Dagny Holle, Charly Gaul, Gregor Broessner

**Affiliations:** 1 Department of Neurology, Headache Outpatient Clinic, Innsbruck Medical University, Anichstrasse 35, 6020 Innsbruck, Austria; 2 Department of Neurology, University of Duisburg-Essen, Essen, Germany

**Keywords:** Migraine, Cluster headache, Tension-type headache, Brain-derived neurotrophic factor

## Abstract

Brain derived neurotrophic factor (BDNF) is associated with pain modulation and central sensitization. Recently, a role of BDNF in migraine and cluster headache pathophysiology has been suspected due to its known interaction with calcitonin gene-related peptide. Bi-center prospective study was done enrolling four diagnostic groups: episodic migraine with and without aura, episodic cluster headache, frequent episodic tension-type headache, and healthy individuals. In migraineurs, venous blood samples were collected twice: outside and during migraine attacks prior to pain medication. In cluster headache patients serum samples were collected in and outside cluster bout. Analysis of BDNF was performed using enzyme-linked immunosorbent assay technique. Migraine patients revealed significantly higher BDNF serum levels during migraine attacks (*n* = 25) compared with headache-free intervals (*n* = 53, *P* < 0.01), patients with tension-type headache (*n* = 6, *P* < 0.05), and healthy controls (*n* = 22, *P* < 0.001). There was no significant difference between patients with migraine with aura compared with those without aura, neither during migraine attacks nor during headache-free periods. Cluster headache patients showed significantly higher BDNF concentrations inside (*n* = 42) and outside cluster bouts (*n* = 24) compared with healthy controls (*P* < 0.01, *P* < 0.05). BDNF is increased during migraine attacks, and in cluster headache, further supporting the involvement of BDNF in the pathophysiology of these primary headaches.

## Introduction

Migraine is a neurologic disorder affecting about 11 % of the population [1]. The mechanisms that have been implicated in the pathophysiology of migraine include neurogenic inflammation, cortical spreading depression, central sensitization, and vascular involvement [2]. Activation of the trigeminovascular [3] system is thought to play a pivotal role in both, migraine and cluster headache pain processing [4]. Various peptides like calcitonin-gene related peptide (CGRP), vasoactive intestinal peptide, and substance P have been shown to mediate nociceptive effects during headache pain generation and central sensitization.

Brain-derived neurotrophic factor (BDNF) is the most abundant neurotrophin within the central and the peripheral nervous system [5]. In addition to effects on neuronal development and differentiation it has been shown to exert a pivotal role in the modulation of pain signaling [6]. It affects plasticity of synapses in trigeminal nociceptive pathways and is expressed in trigeminal ganglion neurons [7–9]. Trigeminal release of BDNF is induced by inflammatory stimuli such as tumor necrosis factor-alpha and vasoactive mediators of neurogenic inflammation including CGRP [8, 10]. CGRP is an important regulator and player in the pathogenesis of migraine and cluster headache through the modulation of nociceptive transmission in the trigeminovascular system [3]. Animal studies show a co-expression of CGRP with BDNF in trigeminal ganglion neurons [7]. Additionally, BDNF release has been shown to be induced by CGRP [9]. Hence interactions between CGRP and BDNF have been suggested to increase migraine susceptibility [11].

This prospective bi-center study aimed to analyze serum levels of BDNF in patients with primary headache disorders, i.e. migraine, cluster headache, and tension-type headache.

## Materials and methods

### Study sites and design

Three groups of headache patients were enrolled in this prospective study: patients with episodic migraine with and without aura, episodic cluster headache, and frequent episodic tension-type headache according to the current criteria of the International Headache Society (ICHD-II [12]). All patients were diagnosed by a headache specialist and recruited in two headache outpatient clinics, respectively: headache outpatient clinic at Innsbruck Medical University, Innsbruck, Austria, and the West German Headache Center at the University of Duisburg-Essen, Essen, Germany. In migraineurs and patients with cluster headache collection of blood samples was performed twice: during migraine attacks and during the interictal phase, inside and outside cluster bouts, respectively. In patients with tension-type headache (outside attack) and healthy controls, one single blood sample was taken. Migraine patients were contacted by phone 3 days after venipuncture during headache-free periods to rule out blood sampling during the premonitory phase of an upcoming migraine attack. Hence, samples of patients, who experienced a migraine attack within 72 h after blood sampling, were excluded from the study.

### Standard protocol approvals, registrations, and patient consents

The study protocol was approved by the institutional review boards at Innsbruck Medical University and the University of Duisburg-Essen (Innsbruck AM3793a, Essen 10-4345). All patients and controls gave written informed consent to study participation prior to study-related procedures.

### Inclusion and exclusion criteria

Patients, males and females, were included if they consented to study participation, and were aged between 18 and 50 years. Migraine patients were included if they suffered from at least one, but no more than eight migraine attacks per month, and had not taken any pain medication including non-steroidal anti inflammatory drugs (NSAIDs), triptans or opioids 48 h prior to blood sample collection and if phone contact was possible 3 days after blood sampling. Patients taking any preventive medication for prophylaxis of migraine or tension-type headache were excluded. Further exclusion criteria were diagnosis of medication overuse headache (according to ICHD II [12]), more than ten headache days per month in migraineurs and patients with tension-type headache and more than 10 days per month with reported intake of medication for the acute treatment of headache. Patients were not included if they had a history of cardio or cerebrovascular disease, diabetes, cancer, severe renal or hepatic disease. Patients with more than one diagnosis of a primary headache, i.e. coexistent migraine and tension-type headache, were excluded from study participation. Pregnancy and breastfeeding were additional exclusion criteria.

### Blood samples and BDNF analysis

All blood samples were processed strictly following an identical predefined protocol in both participating centers. Venous cubital blood samples were centrifuged after a clotting time of 30–60 min at 1,500 rcf for 15 min to obtain serum. Immediately after centrifugation, serum samples were divided into aliquots of 300 μl each and stored at −20 °C until use. Repeated freezing thaw cycles were avoided. Measurement of BDNF was performed using sandwich enzyme-linked immunosorbent assay according to the manufacturer’s instructions (R&D Systems, Minneapolis, Minnesota). Precision of the assay was verified by determination of the inter- and intra-assay coefficients of variation, which were <15 and <10 %, respectively. Patient and control samples were run on the same plates with identical standard curves.

### Statistical analysis

The Kolmogorov–Smirnov test was used to test for normal distribution of continuous variables. To analyze repeated measurements of serum levels the paired *t* test for parametric data was performed and Dunn’s corrected for multiple comparisons. The Mann–Whitney *U* test was performed for comparison between two groups; for more than two comparisons the Kruskal–Wallis test was used. In order to test for important covariates (age, gender, hormonal contraception and smoking status) general estimating equation (GEE) models were calculated. The Pearson correlation was used to test the association between headache days per month/attack frequency and BDNF serum levels. Data are presented as median (interquartile range, IQR) unless otherwise stated. A two-sided *P* value of <0.05 was considered as statistically significant. All statistical analyses were performed using the PASW 18.0 package (SPSS Inc., Chicago, IL, USA) and the GraphPad Prism 5 software (GraphPad Prism Software Inc. San Diego, CA, USA).

## Results

### Baseline characteristics

Fifty-nine patients with episodic migraine were included in the study. Of those 23 patients were diagnosed as migraine with aura (ICHD II [12]). In 19 patients paired samples during and outside migraine attacks were available. A total number of 52 cluster headache patients were included in the analysis. Paired samples, i.e. in versus out bout, were collected in 14 patients with episodic cluster headache. Six patients with tension-type headache in a pain-free interval as well as 22 healthy controls were included. Age, gender, smoking, and hormonal contraception did not have significant effects on peripheral BDNF serum levels (data not shown). For demographic and clinical data of the study population (see Table 1). BDNF serum concentrations are listed in Table 2.

**Table 1:** Demographic and clinical data of the study population

Characteristics	Migraine with aura		Migraine without aura		Cluster headache		Tension-type headache	Healthy controls
	Outside attack	During attack	Outside attack	During attack	Outside bout	Inside bout	Outside attack	Headache free
*N*	22	13	27	8	24	42	6	22
Mean age (±SD)	32.05 (±10.05)		31.48 (±7.89)		39.98 (±13.19)		30.83 (±5.60)	28.63 (±3.88)
Female gender, *n* (%)	17 (77)		20 (74)		7 [13]		5 (83)	10 (45)
Paired, *n*	11		8		14		n.a.	n.a.

*n.a.* not applicable

**Table 2:** Serum concentrations of brain-derived neurotrophic factor (BDNF) in ng/ml

	Migraine		Cluster headache		Tension-type headache	Healthy controls
	Outside attack	During attack	Outside bout	Inside bout	Outside attack	Headache free
BDNF (all patients)	24.50 (±9.17)	31.24 (±9.31)	28.29 (±12.77)	27.77 (±9.35)	20.97 (±2.49)	21.20 (±5.64)
BDNF (paired samples)	31.07 (±10.58)	33.37 (±9.28)	30.29 (±13.13)	29.96 (±9.64)	n.a.	n.a.

All data are presented as mean ± SD

*n.a.* not applicable

### Migraine

**Fig. 1:**
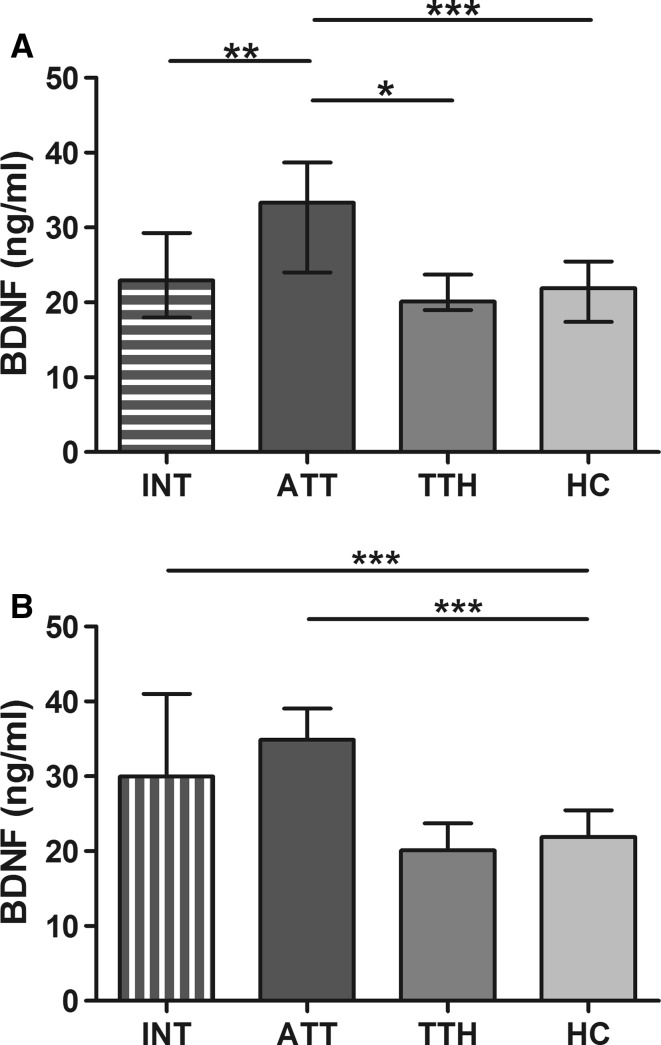
**a** Significant increase of brain-derived neurotrophic factor (*BDNF*) during migraine attacks (*ATT*) compared with headache-free periods (*INT*), tension-type headache (*TTH*), and healthy controls (*HC*). **b** Comparison of* BDNF* levels in 19 patients, for whom paired samples—during migraine attacks (*ATT*) and headache-free periods (*INT*)—were available

**Fig. 2:**
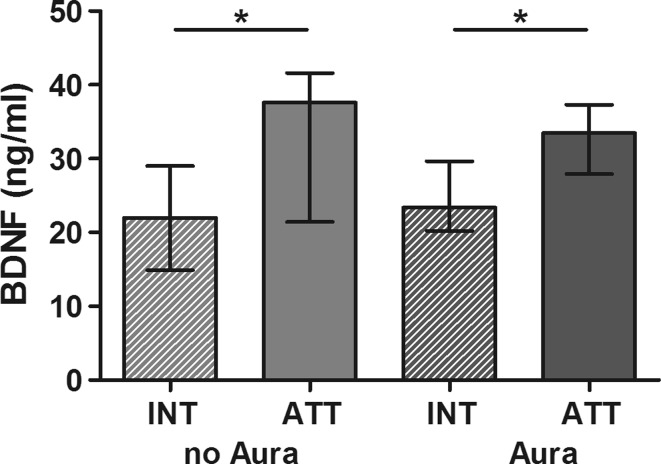
BDNF levels in migraine with and without aura during headache-free periods (*INT*) and migraine attacks (*ATT*), respectively

In migraineurs, BDNF was significantly elevated during migraine attacks compared with headache-free periods (*P* < 0.01), tension-type headache (*P* < 0.05) and healthy controls (*P* < 0.001, Fig. 1a). When comparing paired samples in 19 patients, for whom sera were available during and outside migraine attacks, no statistically significant difference in BDNF concentrations was evident (Fig. 1b). This effect is mainly driven by three patients. When excluding these three patients there is a statistical trend towards higher interictal BDNF levels compared with inside-attack BDNF concentrations (*P* = 0.05). However, both outside-attack as well as ictal BDNF levels were significantly higher compared with controls (*P* < 0.001). A subgroup analysis for migraine with and without aura did not reveal different distribution of BDNF levels (Fig. 2). However, in both groups, migraine with and without aura, BDNF was again significantly elevated during migraine attacks compared with interictal levels (*P* < 0.05, respectively). No statistically significant correlation with migraine attack frequency/headache days per month (*r* = 0.006), headache duration (*r* = 0.14) and serum concentrations of BDNF was found.

### Cluster headache

**Fig. 3:**
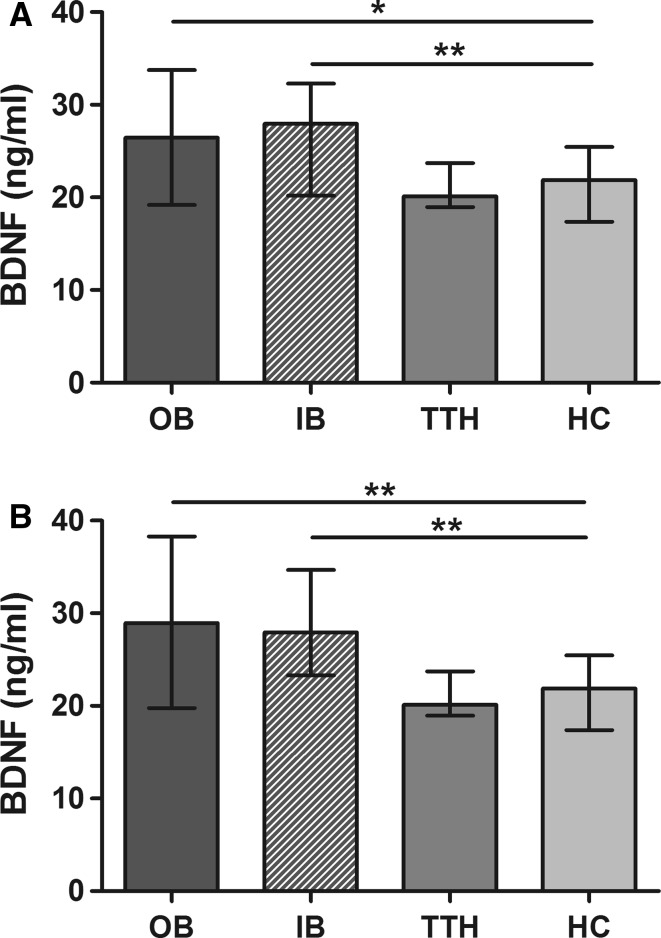
**a** Elevated serum levels of brain-derived neurotrophic factor (*BDNF*) inside (*IB*) and outside cluster bouts (*OB*). **b** Paired samples of cluster headache patients (*n* = 14) show a similar distribution with a significant elevation of BDNF levels in and out cluster bouts compared with controls

BDNF was increased outside cluster bouts as well as during cluster bouts compared with healthy control subjects (*P* < 0.05 and *P* < 0.01, respectively, Fig. 3a). When comparing paired blood samples in patients with cluster headache, BDNF was significantly elevated inside and outside bouts compared with patients with tension-type headache, and healthy controls (*P* < 0.01, *P* < 0.05, Fig. 3b. In three patients with cluster headache serum was obtained during cluster attacks. In those patients BDNF concentrations did not emerge different from in bout levels (data not shown).

There was no significant difference in BDNF levels between patients with tension-type headache and healthy controls (*P* = 0.84).

## Discussion

In this prospective bi-center study, BDNF serum levels in patients with episodic migraine with and without aura, episodic cluster headache, episodic tension-type headache, and healthy control subjects were analyzed. Our main findings were (1) BDNF is significantly elevated in patients with migraine and cluster headache compared with patients with tension-type headache and healthy controls. (2) During migraine attacks BDNF serum levels are significantly higher compared with headache-free periods and controls. This effect also holds true in both migraine subpopulations, with and without aura, respectively. (3) Patients with cluster headache revealed significantly increased BDNF during cluster bouts, but showed also elevated out-bout levels of BDNF.

BDNF is a member of the neurotrophin family and has been recognized as an important modulator of nociceptive pathways [13]. However, the effects of BDNF within the nociceptive system seem to be manifold. An anti-nociceptive effect is suggested in central pathways [14–16]. This is supported by results from animal studies showing analgesia after intracerebroventricular administration of BDNF [14]. By contrast, allodynia is mediated by BDNF in experimental models of neuropathic pain [17]. These contradictory results are partly explained by dose-dependent effects of BDNF with low doses causing hyperalgesia, whereas higher doses may lead to analgesia, an effect that might be induced by the activation of different intracellular pathways [13]. The trigeminal system is supposed to play a central role not only in migraine but also in cluster headache pathology [2, 3]. In vitro studies have demonstrated the expression of BDNF in trigeminal ganglion neurons [6]. BDNF release is induced by trigeminal stimulation and nociceptive inputs [18]. Interestingly, BDNF is co-expressed with CGRP in trigeminal ganglion neurons [9]. CGRP is one of the key molecules in migraine and cluster headache pathogenesis [4, 19]. It is released after activation of perivascular sensory trigeminal nerve fibers [20]. Levels of CGRP measured in the external jugular vein have been found to be elevated during migraine attacks and cluster headache [21]. Interactions between neurotrophic factors and neuropeptides such as CGRP are manifold: BDNF release from trigeminal ganglion neurons has been shown to be induced by CGRP in vitro, an effect that was reversible in the presence of a CGRP-receptor antagonist [8]. Interestingly, CGRP gene expression is increased by the nerve growth factor NGF via activation of CGRP promotor enhancers [22]. The application of an anti-BDNF antibody decreased both CGRP and BDNF in rat dorsal root ganglia [23]. Results of a recent case–control study suggest an interaction of CGRP and BDNF polymorphisms, contributing to migraine susceptibility [11].

Neurogenic inflammation is thought to play a pivotal role in migraine pathology [24, 25]. Interestingly, BDNF is up-regulated in primary sensory neurons by inflammation [10, 26]. Peripheral and cerebrospinal fluid levels of pro-inflammatory cytokines have been found to be elevated in migraineurs [27–29]. Cytokine-induced release of pain modulators such as BDNF from trigeminal neurons might underline the interaction of inflammatory and neuronal pathways leading to neurogenic inflammation.

This study is limited by the fact that we could not find a statistical significant difference between interval and attack BDNF values in the subgroup of the paired samples. When interpreting these findings one has to keep in mind that this result is driven by three patients only. Thus, excluding these three patients reaches again statistical significance, confirming the results of the whole group analysis. Still this cannot be simply regarded as a selection bias and should be further investigated in a future study. Our study supports the hypothesis that BDNF has an important role in migraine pathophysiology. Significant elevation during migraine attacks even in serum samples from peripheral, cubital venipuncture could be shown. BDNF increase might be interpreted as a general reaction to pain. However, the elevation of BDNF even outside migraine attacks (in the subgroup analysis of patients with paired samples) and also outside cluster bouts without presence of pain in contrast to normal levels of BDNF in tension-type headache suggests an exclusive effect in headaches with trigeminal involvement.

Cortical spreading depression, characterized by a wave of oligemia spreading along the cortex of the brain, has been considered as the pathophysiological equivalent of migraine aura [30, 31]. Up-regulation of BDNF mRNA has been observed after cortical spreading depression in experimental in vivo studies [32, 33]. However, we did not find a difference in BDNF serum concentrations between patients with migraine with and without aura.

Our findings are in contrast with the results of Blandini et al. [34] reporting decreased platelet levels of BDNF in patients with migraine with and without aura as well as patients with cluster headache. By contrast, circulating BDNF serum levels measured in our study are elevated in migraineurs. These conflicting results might be attributable to platelet activation during migraine attacks with immediate release of BDNF upon activation leading to decreased platelet levels [35]. In a prospective pilot study BDNF serum levels were analyzed in nine migraineurs during migraine attacks and interictal phase [27]. Consistent with our results Tanure et al. [27] report an elevation of BDNF during migraine attacks compared with the headache-free period.

The present study is the first to report an increase of BDNF in patients with cluster headache. Surprisingly, elevated BDNF levels were detected not only during cluster bouts but also outside bouts. Increased CGRP levels were reported in cluster headache patients in jugular venous blood samples, indicating trigeminal activation [36]. Since BDNF is co-expressed with CGRP in trigeminal ganglion neurons, increased BDNF levels might indicate trigeminal activation [8, 9]. In a study by Di Piero et al. [37] pain stimulation in cluster patients outside bouts revealed a pathological response in cerebral blood flow compared with healthy controls suggesting that alterations in pain processing are also present outside active cluster periods. Elevation of BDNF during and outside cluster bouts might result from continuous trigeminal activation in cluster headache patients, which should be investigated in future trials.

This study is limited by the small size of the patient group with frequent episodic tension-type headache. However, all patients were recruited from two specialized headache outpatient clinics, where the patient population is rather focused on chronic but on episodic tension-type headache. Moreover, many patients with tension-type headache seen in specialized headache centers suffer from medication overuse headache and were thus not eligible for study participation according to the predefined study protocol. We were not able to include a higher number of patients with cluster headache during cluster attacks. This was mainly caused by the fact that cluster attacks showed an average duration of 75 min and face-to-face patient contact was often not possible within that time period.

Various studies suggest the involvement of BDNF in pain processing and peripheral as well as central sensitization [38, 39]. This is the first study to show elevation of BDNF in both migraine and cluster patients. Our results underline the important role of this neurotrophic factor in nociceptive pathways.
